# Supplementary material for: Paranoid thinking and perceived competitive intention
Source: PeerJ. 2023 Mar 10;11:e15003. doi: 10.7717/peerj.15003 (PMC10010176; doi:10.7717/peerj.15003)
Supplement: Supplemental Information 1 [file peerj-11-15003-s001.pdf]

Supplementary Information for "Paranoid thinking and perceived competitive intention."

Yutaka Horita[1]
1. Department of Psychology, Teikyo University, Tokyo, Japan

Supplementary Figures

Figure S1: Distributions of paranoia scores (R-GPTS Persecution) shown separately by each role.

Figure S2: Distributions of the Dictators' paranoia scores according to their decisions in the Dictator Game.

Figure S3: Distributions of supposition regarding the Dictators' self-interest by the Recipients' paranoia levels.

Supplementary Tables

Table S1: Results of the ordinal logistic regression models for predicting Recipients' expectation that Dictators would choose the competitive allocation.

Table S2: Results of the ordinal logistic regression models for predicting the Recipient's supposition that Dictators would have harmful intentions.

Table S3: Results of the logistic regression model for predicting Recipient's decision to avoid the allocation by Dictators.

Table S4: Results of the logistic regression model for predicting the Dictator's decision to choose a competitive option.

Table S5: Results of the ordinal logistic regression model for predicting the Recipient's supposition of the Dictators' self-interest.

Table S6: Results of a logistic regression model for predicting Recipient's decision to avoid the allocation by Dictators using all obtained variables as predictors.

Table S7: Results of the linear regression model for predicting Recipients' expectation that Dictators would choose the competitive allocation.

Table S8: Results of the linear regression model for predicting the Recipient's supposition that Dictators would have harmful intentions.

Table S9: Results of the linear regression model for predicting the Recipient's supposition of the Dictators' self-interest.

Supplementary Materials

- Instructions for pre-survey
  - Questionnaire
- Instructions for Dictator game
  - Instructions for Dictators
  - Instructions for Recipients

Supplementary Reference

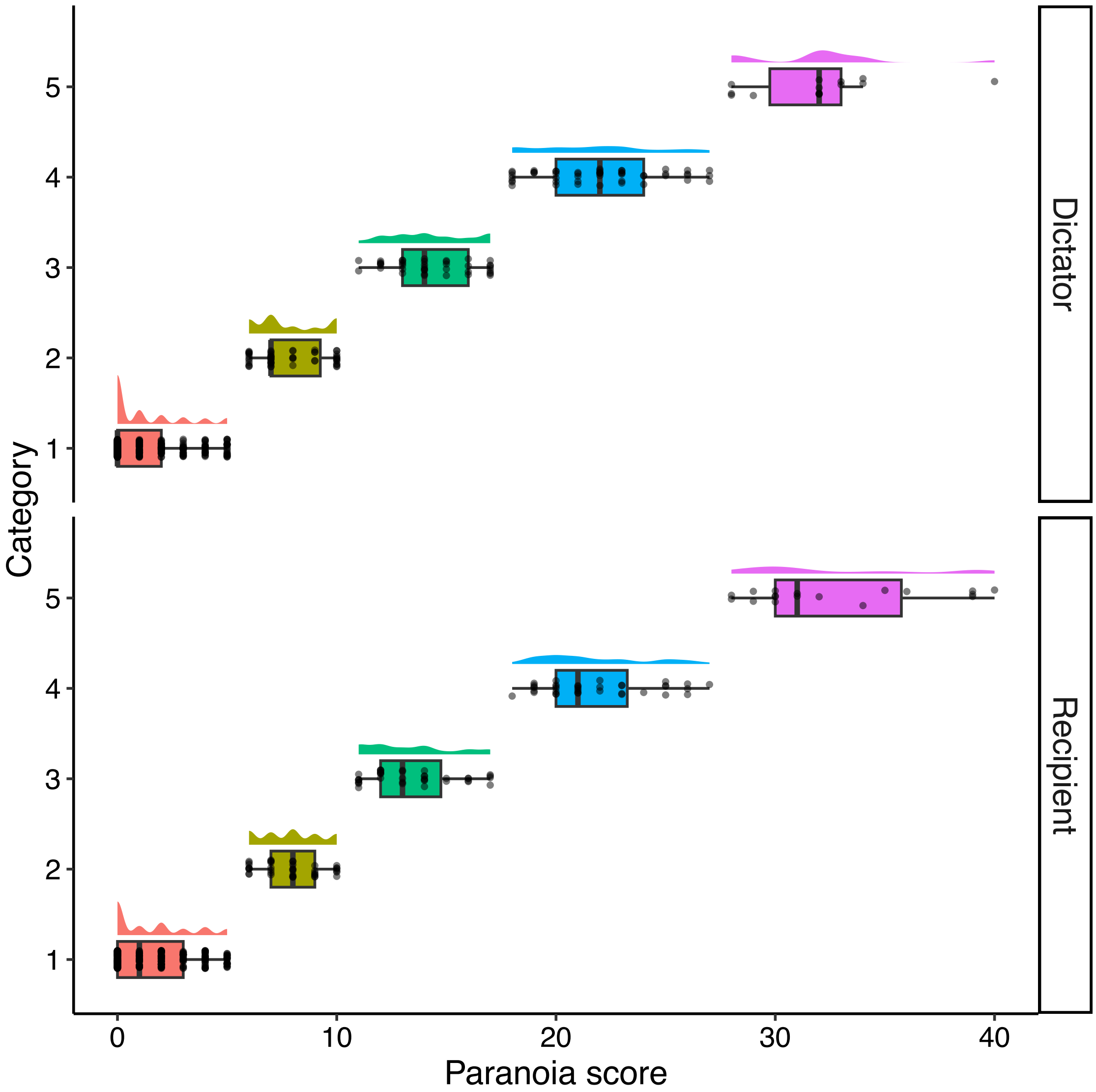

Supplementary Figure S1: Distributions of paranoia scores (R-GPTS Persecution) shown separately by each role.

Each point represents each participant, and random vertical jitter was added to each point for ease of visibility. The box, the thick line in each box, and the whisker represent the interquartile range (IQR), the median, and the distances 1.5 × IQR, respectively.

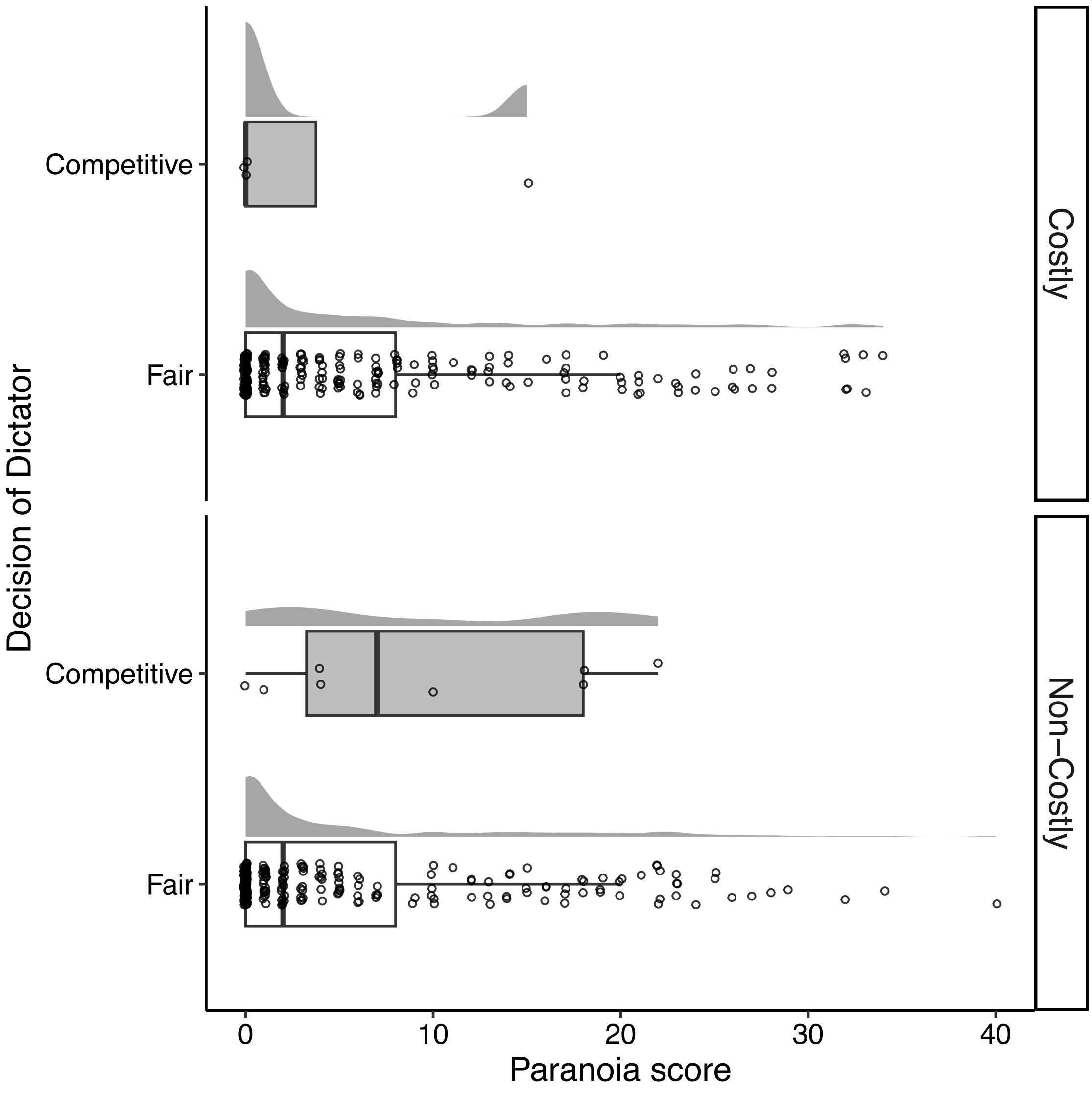

Supplementary Figure S2: Distributions of the Dictators' paranoia scores according to their decisions in the Dictator Game.

Each point represents each Dictator, and random vertical jitter was added to each point for ease of visibility. The box, the thick line in each box, and the whisker represent the IQR, the median, and the distances 1.5 × IQR, respectively.

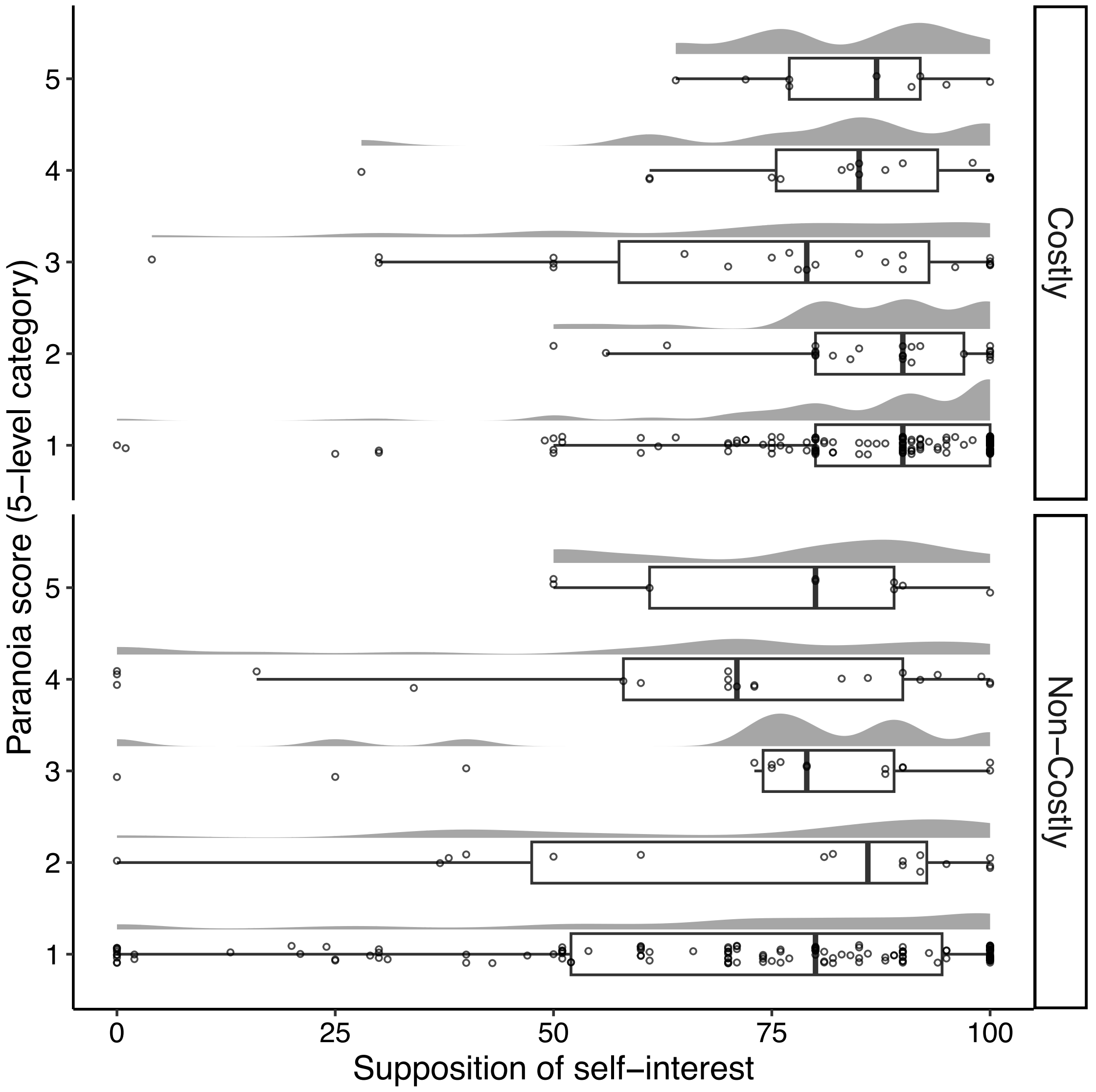

Supplementary Figure S3: Distributions of supposition regarding the Dictators' self-interest by the Recipients' paranoia levels.

Each point represents each Recipient, and random vertical jitter was added to each point for ease of visibility. Boxplots indicate the distributions of the paranoia score. The box, the thick line in each box, and the whisker represent the interquartile range (IQR), the median, and the distances 1.5 × IQR, respectively.

Supplementary Table S1: Results of the ordinal logistic regression models for predicting Recipients' expectation that Dictators would choose the competitive allocation.

Expectation score was coded as a five-level ordinal variable. Each estimate represents the mean of the posterior distribution of the parameter. SD: Standard deviation, CI: Credible intervals.

Expectation			
Parameters	Estimates	SD	[95% CI]
Intercept 1|2	–0.47	0.10	[–0.68, –0.27]
Intercept 2|3	0.53	0.11	[0.32, 0.73]
Intercept 3|4	1.09	0.12	[0.87, 1.32]
Intercept 4|5	2.36	0.17	[2.03, 2.71]
Paranoia	0.43	0.09	[0.24, 0.61]
Condition (Costly = 1)	–0.20	0.18	[–0.56, 0.15]
Gender (Male = 1)	–0.08	0.20	[–0.47, 0.31]
Age	0.07	0.09	[–0.11, 0.26]
Paranoia × Condition	0.19	0.18	[–0.16, 0.54]

Supplementary Table S2: Results of the ordinal logistic regression models for predicting the Recipient's supposition that Dictators would have harmful intentions.

Harmful intent score was coded as a five-level ordinal variable. Each estimate represents the mean of the posterior distribution of the parameter. SD: Standard deviation, CI: Credible intervals.

Harmful intention			
Parameters	Estimates	SD	[95% CI]
Intercept 1|2	-0.23×10^{-2}	0.10	[–0.21, 0.20]
Intercept 2|3	0.81	0.11	[0.59, 1.03]
Intercept 3|4	1.40	0.13	[1.15, 1.65]
Intercept 4|5	2.30	0.17	[1.98, 2.63]
Paranoia	0.50	0.09	[0.32, 0.69]
Condition (Costly = 1)	–0.50	0.19	[–0.88, –0.13]
Gender (Male = 1)	–0.50	0.21	[–0.91, –0.09]
Age	–0.05	0.10	[–0.24, 0.14]
Paranoia × Condition	0.25	0.18	[–0.10, 0.60]

Supplementary Table S3: Results of the logistic regression model for predicting Recipient's decision to avoid the allocation by Dictators.

The decision of the Recipient was coded as a binary value (0 = dictators' allocation, 1 = avoidance). Each estimate represents the mean of the posterior distribution of the parameter. SD: Standard deviation, CI: Credible intervals.

Avoidance			
Parameters	Estimates	SD	[95% CI]
Intercept	–0.93	0.11	[−1.15, –0.71]
Paranoia	0.01	0.12	[−0.22, 0.24]
Condition (Costly = 1)	–0.32	0.22	[−0.76, 0.12]
Gender (Male = 1)	0.08	0.24	[−0.40, 0.55]
Age	0.03	0.11	[−0.19, 0.25]
Paranoia × Condition	–0.19	0.23	[−0.64, 0.25]

Supplementary Table S4: Results of the logistic regression model for predicting the Dictator's decision to choose a competitive option.

Dictator's decision was coded as a binary value (0 = fair, 1 = competitive). Each estimate represents the mean of the posterior distribution of the parameter. SD: Standard deviation, CI: Credible interval.

Competitive allocation			
Parameters	Estimates	SD	[95% CI]
Intercept	−4.75	0.58	[−6.03, −3.78]
Paranoia	−0.11	0.47	[−1.21, 0.65]
Condition (Costly = 1)	−1.05	0.77	[−2.69, 0.35]
Gender (Male = 1)	2.35	0.89	[0.81, 4.30]
Age	0.46	0.30	[−0.14, 1.05]
Paranoia × Condition	−1.16	0.92	[−3.28, 0.31]

Supplementary Table S5: Results of the ordinal logistic regression model for predicting the Recipient's supposition of the Dictators' self-interest.

The self-interest score was coded as a five-level ordinal variable. Each estimate represents the mean of the posterior distribution of the parameter. SD: Standard deviation, CI: Credible interval.

Self-interest			
Parameters	Estimates	SD	[95% CI]
Intercept 1|2	−2.96	0.22	[−3.42, −2.55]
Intercept 2|3	−2.16	0.16	[−2.48, −1.85]
Intercept 3|4	−1.42	0.13	[−1.67, −1.17]
Intercept 4|5	−0.20	0.10	[−0.41, -0.21×10^{-2}]
Paranoia	−0.08	0.10	[−0.27, 0.11]
Condition (Costly = 1)	0.95	0.20	[0.57, 1.34]
Gender (Male = 1)	−0.05	0.21	[−0.47, 0.36]
Age	−0.01	0.10	[−0.21, 0.18]
Paranoia × Condition	−0.14	0.19	[−0.51, 0.23]

Supplementary Table S6: Results of a logistic regression model for predicting Recipient's decision to avoid the allocation by Dictators using all obtained variables as predictors.

Recipient 's decision was coded as a binary value (0 = dictator allocation, 1 = avoidance). All obtained variables were included as predictors. Each estimate represents the mean of the posterior distribution of the parameter. SD: Standard deviation, CI: Credible interval.

Avoidance			
Parameters	Estimates	SD	[95% CI]
Intercept	−1.07	0.13	[−1.32, −0.83]
Paranoia	−0.21	0.13	[−0.48, 0.04]
Condition (Costly = 1)	−0.34	0.25	[−0.83, 0.14]
Gender (Male = 1)	0.20	0.26	[−0.32, 0.72]
Age	0.34×10^{-2}	0.13	[−0.25, 0.25]
Expectation	0.72	0.13	[0.47, 0.98]
Harmful intent	0.27	0.13	[0.02, 0.53]
Self-interest	0.23	0.14	[−0.04, 0.51]
Paranoia × Condition	−0.27	0.24	[−0.74, 0.20]

Supplementary Table S7: Results of the linear regression models for predicting Recipient's expectation that Dictators would choose the competitive allocation. Expectation score was standardized. Each estimate represents the mean of the posterior distribution of the parameter. SD: Standard deviation, CI: Credible intervals.

Expectation			
Parameters	Estimates	SD	[95% CI]
Intercept	0.43×10^{-3}	0.05	[−0.10, 0.10]
Paranoia	0.22	0.05	[0.12, 0.32]
Condition (Costly = 1)	–0.11	0.10	[–0.30, 0.09]
Gender (Male = 1)	−0.06	0.11	[−0.27, 0.15]
Age	0.04	0.05	[−0.05, 0.14]
Paranoia × Condition	0.07	0.10	[−0.12, 0.27]

Supplementary Table S8: Results of the linear regression models for predicting Recipient's supposition that Dictators would have harmful intentions by each condition.

Harmful intent score was standardized. Each estimate represents the mean of the posterior distribution of the parameter. SD: Standard deviation, CI: Credible intervals.

Harmful intention			
Parameters	Estimates	SD	[95% CI]
Intercept	0.63×10^{-3}	0.05	[−0.09, 0.09]
Paranoia	0.26	0.05	[0.17, 0.36]
Condition (Costly = 1)	–0.24	0.10	[−0.43, –0.05]
Gender (Male = 1)	−0.24	0.10	[−0.45, –0.04]
Age	−0.02	0.05	[−0.12, 0.07]
Paranoia × Condition	0.11	0.10	[−0.07, 0.30]

Supplementary Table S9: Results of the linear regression model for predicting the Recipient's supposition of the Dictators' self-interest.

Self-interest score was standardized. Each estimate represents the mean of the posterior distribution of the parameter. SD: Standard deviation, CI: Credible interval.

Self-interest			
Parameters	Estimates	SD	[95% CI]
Intercept	-0.50×10^{-3}	0.05	[−0.09, 0.09]
Paranoia	−0.04	0.05	[−0.14, 0.06]
Condition (Costly = 1)	0.53	0.10	[0.34, 0.72]
Gender (Male = 1)	0.01	0.11	[−0.19, 0.22]
Age	−0.01	0.05	[−0.11, 0.08]
Paranoia × Condition	−0.08	0.10	[−0.27, 0.11]

Supplementary Materials

Instructions for pre-survey

Questionnaire

(R-GPTS: Freeman et al., 2021)

Please read each of the following statements carefully. They refer to thoughts and feelings you may have had about others **over the last month**. Think about the last month and indicate the extent of these feelings from **0 (Not at all) to 4 (Totally)**.
Please complete both Part A and Part B.
(N.B. Please do not rate items according to any experiences you may have had under the influence of drugs.)

Part A

	Not at all (0)	1	2	Totally (4)
1. I spent time thinking about friends gossiping about me.	○	○	○	○
2. I often heard people referring to me.	○	○	○	○
3. I have been upset by friends and colleagues judging me critically.	○	○	○	○
4. People definitely laughed at me behind my back.	○	○	○	○
5. I have been thinking a lot about people avoiding me.	○	○	○	○
6. People have been dropping hints for me.	○	○	○	○
7. I believed that certain people were not what they seemed.	○	○	○	○
8. People talking about me behind my back upset me.	○	○	○	○

Please read each of the following statements carefully. They refer to thoughts and feelings you may have had about others **over the last month**. Think about the last month and indicate the extent of these feelings from **0 (Not at all) to 4 (Totally)**.
Please complete both Part A and Part B.
(N.B. Please do not rate items according to any experiences you may have had under the influence of drugs.)

Part B

	Not at all (0)	1	2	Totally (4)
1. Certain individuals have had it in for me.	○	○	○	○
2. People wanted me to feel threatened, so they stared at me.	○	○	○	○
3. I was certain people did things in order to annoy me.	○	○	○	○
4. I was convinced there was a conspiracy against me.	○	○	○	○
5. I was sure someone wanted to hurt me.	○	○	○	○
6. I couldn't stop thinking about people wanting to confuse me.	○	○	○	○
7. I was distressed by being persecuted.	○	○	○	○
8. It was difficult to stop thinking about people wanting to make me feel bad.	○	○	○	○
9. People have been hostile towards me on purpose.	○	○	○	○
10. I was angry that someone wanted to hurt me.	○	○	○	○

What is your gender?

○ Male
○ Female
○ Prefer not to say

What is your age?

____

Have you ever participated in a survey similar to this one before?

	0: I have never participated in a similar one.	1	2	3	4. I have participated in a very similar one.
Please indicate to what extent.	◯	◯	◯	◯	◯

Instructions for Dictator game

<u>Instructions for Dictators</u>

Individuals participating in the study will receive $0.60 as remuneration. In addition, the amount of money earned from this experiment will be paid as a bonus.

You will be randomly paired with another participant.

In this experiment, there are two roles: Player 1 and Player 2.
You are assigned to the role of Player 1.

Player 1 decides how to allocate the money between Player 1 and Player 2.

You choose either option A or B.
A: You will receive $0.50, and Player 2 will receive $0.50.
B: You will receive $0.40, and Player 2 will receive $0.10.
(B: You will receive $0.50, and Player 2 will receive $0.10.)

This experiment will be carried out **just once**.

To confirm whether you have understood the rules of the experiment or not, we will ask you some questions.

Comprehension questions

1. If you choose <u>option A</u>, how much money will each of you and Player 2 receive?

You:
- ○ $0.40
- ○ $0.50

(Correct answer is $0.50.)

Player 2:
- ○ $0.10
- ○ $0.50

(Correct answer is $0.50.)

If you choose <u>option B</u>, how much money will each of you and Player 2 receive?

You:

- ◯ $0.40
- ◯ $0.50

(In the costly condition, correct answer is $0.40. In the non-costly condition, correct answer is $0.50)

Player 2:

- ◯ $0.10
- ◯ $0.50

(Correct answer is $0.10.)

If you answer all questions correctly, you can proceed to the Decision Screen. Please note that you cannot proceed to the following pages and receive your bonus if you do not answer all questions correctly.

Please indicate your decision by selecting either option A or B.

- ◯ A: I will receive $0.50, and Player 2 will receive $0.50.
- ◯ B: I will receive $0.40, and Player 2 will receive $0.10.

(B: I will receive $0.50, and Player 2 will receive $0.10.)

As explained in the instructions, you will be paired with one of the other participants. All participants are real. Nevertheless, we will ask you a question to be sure.

Please indicate to what extent you believed that the other player really existed.

	0: Very skeptical that the others were real.	1	2	3	4: Very confident that the others were real.
Please choose one.	◯	◯	◯	◯	◯

Have you ever participated in an experiment similar to this one before?

	0: I have never participated in a similar one.	1	2	3	4. I have participated in a very similar one.
Please choose one.	○	○	○	○	○

Instructions for Recipients

Individuals participating in the study will receive $0.60 as remuneration. In addition, the amount of money earned from this experiment will be paid as a bonus.

You will be randomly paired with another participant.

In this experiment, there are two roles: Player 1 and Player 2.
You are assigned to the role of Player 2.

This experiment is composed of the following two steps.

Step 1:
At first, Player 1 made their decision.
Player 1 chose either option A or B.

A: They receive $0.50, and Player 2 receives $0.50.
B: They receive $0.40, and Player 2 receives $0.10.
(B: They receive $0.50, and Player 2 receives $0.10.)

Step 2:
You choose either option X or Y without being informed of Player 1's decision.

X: You will receive the money according to Player 1's decision.
You will receive either $0.50 or $0.10.

Y: You will receive $0.30 for sure.
You will not receive the money that Player 1 decided to give you.

We have already collected all decisions made by Player 1. Player 1 received the money allocated to themselves. **Player 1 was not informed that Player 2 would make a decision**.

This experiment will be carried out **just once**.

After collecting all data, we will randomly make pairs of participants, and this random pairing process will determine your bonus.

To confirm whether you have understood the rules of the experiment or not, we will ask you some questions.

Comprehension questions

1. Suppose you choose option X. If Player 1 had chosen option A, how much money would each of you and Player 1 receive?

Player 1:

- ◯ $0.40
- ◯ $0.50

(Correct answer is $0.50.)

You:

- ◯ $0.10
- ◯ $0.30
- ◯ $0.50

(Correct answer is $0.50.)

2. Suppose you choose option X. If Player 1 had chosen option B, how much money would each of you and Player 1 receive?

Player 1:

- ◯ $0.40
- ◯ $0.50

(In the costly condition, correct answer is $0.40. In the non-costly condition, correct answer is $0.50)

You:

- ◯ $0.10
- ◯ $0.30
- ◯ $0.50

(Correct answer is $0.10.)

3. If you choose option Y, how much money will you receive?

You:

- ○ $0.10
- ○ $0.30
- ○ $0.50

(Correct answer is $0.30.)

4. Did Player 1 know that you would choose either option X or Y?

- ○ They knew it.
- ○ They did not know it.

(Correct answer is "They did not know it".)

If you answer all questions correctly, you can proceed to the Decision Screen. Please note that you cannot proceed to the following pages and receive your bonus if you do not answer all questions correctly.

Player 1 has chosen either A or B.

A: They receive $0.50, and Player 2 receives $0.50.
B: They receive $0.40, and Player 2 receives $0.10.
(B: They receive $0.50, and Player 2 receives $0.10.)

Please indicate your decision by selecting either option X or Y.

- ○ X: I will receive the money according to Player 1's decision.
- ○ Y: I will receive $0.30 for sure.

The participants assigned to Player 1 have already finished their decisions.

A: They receive $0.50, and Player 2 receives $0.50.

B: They receive $0.40, and Player 2 receives $0.10.

(B: They receive $0.50, and Player 2 receives $0.10.)

How many of them do you think chose **option B**?

Please use the slider below to indicate the **percentage** of participants in Player 1 who

would have chosen option B.

	0 10 20 30 40 50 60 70 80 90 100
The percentage (%)	

Please use the slider below to indicate the extent to which you believe Player 1's decisions were driven by their **desire to earn money** in the experiment.
(0: not at all - 100: completely)

	0 10 20 30 40 50 60 70 80 90 100
The extent	

Please use the slider below to indicate to what extent Player 1's decisions were driven by their **desire to reduce Player 2's bonus** in the experiment.

(0: not at all - 100: completely)

	0 10 20 30 40 50 60 70 80 90 100
The extent	

As explained in the instructions, you will be paired with one of the other participants. All participants are real. Nevertheless, we will ask you a question to be sure.

Please indicate to what extent you believed that the other player really existed.

	0: Very skeptical that the others were real.	1	2	3	4: Very confident that the others were real.
Please choose one.	○	○	○	○	○

Have you ever participated in an experiment similar to this one before?

	0: I have never participated in a similar one.	1	2	3	4. I have participated in a very similar one.
Please choose one.	○	○	○	○	○

Supplementary Reference

Freeman D, Loe BS, Kingdon D, Startup H, Molodynski A, Rosebrock L, Brown P, Sheaves B, Waite F, Bird JC. 2021. The revised Green et al., Paranoid Thoughts Scale (R-GPTS): Psychometric properties, severity ranges, and clinical cut-offs. *Psychological Medicine* 51: 244–253. DOI: 10.1017/S0033291719003155
